# Low dose amiodarone reduces tumor growth and angiogenesis

**DOI:** 10.1038/s41598-020-75142-1

**Published:** 2020-10-22

**Authors:** Eliana Steinberg, Arnon Fluksman, Chalom Zemmour, Katerina Tischenko, Adi Karsch-Bluman, Yifat Brill-Karniely, Amy E. Birsner, Robert J. D’Amato, Ofra Benny

**Affiliations:** 1 The Institute for Drug Research, The School of Pharmacy, Faculty of Medicine, The Hebrew University of Jerusalem, Jerusalem, Israel; 2 Vascular Biology Program, Department of Surgery, Boston Children’s Hospital, Harvard Medical School, Boston, MA USA; 3 Department of Ophthalmology, Boston Children’s Hospital, Harvard Medical School, Boston, MA USA

**Keywords:** Biochemistry, Cancer, Cell biology, Diseases, Cancer

## Abstract

Amiodarone is an anti-arrhythmic drug that was approved by the US Food and Drug Administration (FDA) in 1985. Pre-clinical studies suggest that Amiodarone induces cytotoxicity in several types of cancer cells, thus making it a potential candidate for use as an anti-cancer treatment. However, it is also known to cause a variety of severe side effects. We hypothesized that in addition to the cytotoxic effects observed in cancer cells Amiodarone also has an indirect effect on angiogensis, a key factor in the tumor microenvironment. In this study, we examined Amiodarone's effects on a murine tumor model comprised of U-87 MG glioblastoma multiforme (GBM) cells, known to form highly vascularized tumors. We performed several in vitro assays using tumor and endothelial cells, along with in vivo assays utilizing three murine models. Low dose Amiodarone markedly reduced the size of GBM xenograft tumors and displayed a strong anti-angiogenic effect, suggesting dual cancer fighting properties. Our findings lay the ground for further research of Amiodarone as a possible clinical agent that, used in safe doses, maintains its dual properties while averting the drug’s harmful side effects.

## Introduction

Amiodarone is a well-known class III anti-arrhythmic drug that has been in clinical use since its introduction into the market more than three decades ago. The drug was approved by the FDA in 1985 for the treatment of ventricular tachyarrhythmias and atrial fibrillation. It inhibits various ion channels, including calcium, sodium and potassium channels, making it a potent drug for treating serious anti-arrhythmic cardiac diseases^1–5^.

Pre-clinical studies suggest that Amiodarone can reverse multidrug resistance in several types of cancers and enhance cytotoxicity effects both as a single agent or in combination with other anti-cancer drugs^6–9^, thus making it a potential candidate for anti-cancer treatment. However, Amiodarone also has an unfavorable toxicity profile affecting a number of organs. Due to its high lipophilicity, Amiodarone accumulates mainly in adipose tissue or highly perfused organs such as the liver, lungs and skin, and its effects may last for months after cessation due to a long biological half-life of up to 100 days^10–12^. The gulf between Amiodarone’s anti-cancer potential and its known toxicity profile presents a sizeable challenge to repurposing the drug. In this study, we chose to examine Amiodarone’s activity on a human brain cancer cell line, U-87 MG, a known cancer model used to form aggressive and angiogenic tumor xenografts^13^.

Our aim was to investigate whether low dose Amiodarone possessed anti-angiogenic properties in addition to its heretofore reported anti-cancer activity. We also wanted to determine whether both these effects are maintained in doses that were substantially lower than the equivalent standard therapeutic levels for treating cardiac conditions^14^.

Data from our in vitro assays, using U-87 MG and endothelial cells, confirmed Amiodarone's strong anti-cancer effects and, for the first time, revealed its significant anti-angiogenic effects. The well-established angiogenesis in vivo models — the Matrigel plug assay and the corneal micropocket assay — showed that low dose Amiodarone, locally or systemically administered, markedly reduced blood vessel formation in addition to significantly suppressing GBM xenograft tumor growth associated with low tumor vascularization.

We propose that low dose Amiodarone possesses both anti-cancer and anti-angiogenic activity that should be explored further as a potential candidate for combating highly vascularized tumors.

## Results

### Amiodarone affects viability, proliferation and mobility of U-87 MG cells

**Figure 1:**
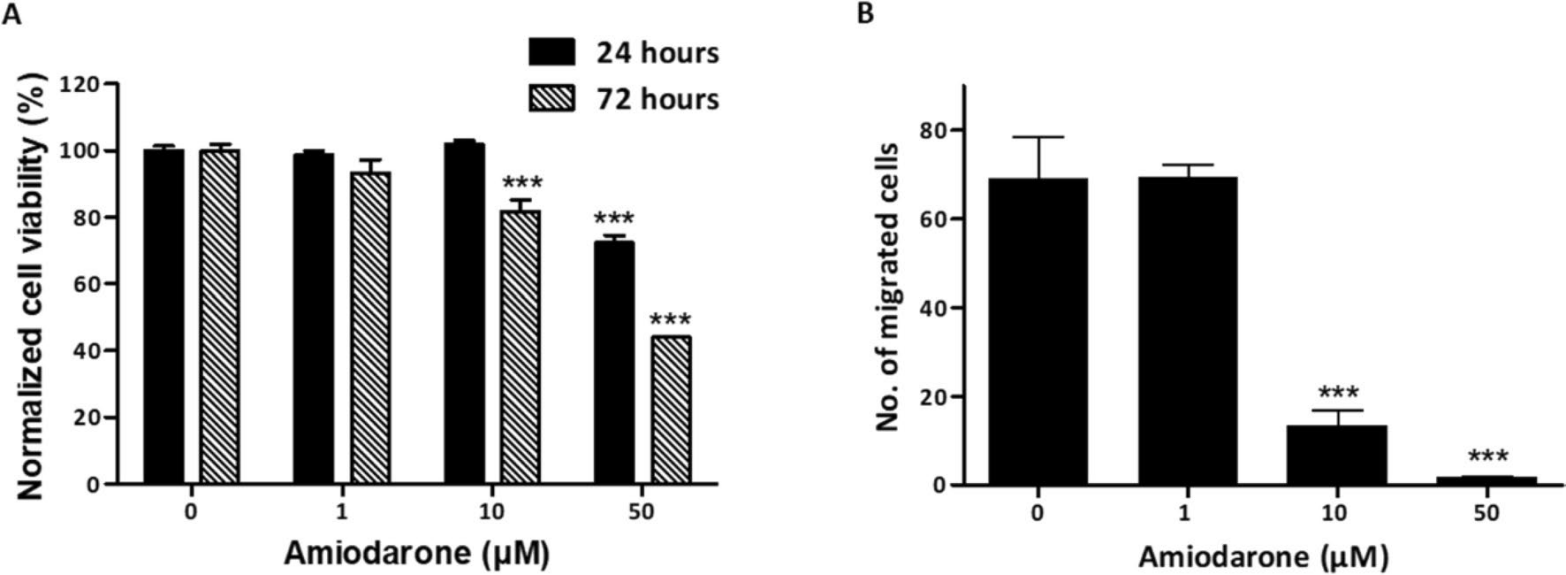
Amiodarone affects U-87 MG cell function. (**A**) Viability assay of U-87 MG cells using WST8, after 24 or 72 h of incubation with ranging concentrations of Amiodarone (0, 1, 10 and 50 μM), investigating cytotoxic and proliferation-rate effects, respectively. n = 9–10. ***p < 0.001, compared with non-treated control cells with the same incubation time. (**B**) Transwell migration assay of U-87 MG cells after 18 h of incubation with ranging concentrations of Amiodarone (0, 1, 10, and 50 μM). Inverted microscope used to detect and count cells on the lower side of inserts' filter (five different fields per well) and average number of migrated cells calculated, n = 6. ***p < 0.001, compared with non-treated control cells. Results are presented as mean ± SEM. Figure supplement 1. Amiodarone’s effect on U-87 MG cell viability.

Viability assays were carried out on U-87 MG cells, human primary GBM cells, after either 24 or 72 h of incubation with Amiodarone, to investigate cytotoxic and proliferation-rate effects, respectively. Amiodarone induced a mild cytotoxic effect with 50 μM, the highest treatment concentration used in our in vitro experiments, reducing the viability of the cells by 28% after 24 h. However, a reduction in the proliferation rate after 72 h already occurred with 10 μM, where 10 and 50 µM treatments led to 18% and 56% reduction, respectively (Fig. 1A). In addition, flow cytometry analyses of Annexin-V-APC and Propidium iodide (PI) staining of U-87 MG cells revealed Amiodarone's effect of reducing cell viability (> 85% with 50 µM), mainly via necrosis, in a dose-dependent manner (Fig. 1–figure supplement 1). A transwell migration assay was conducted to explore Amiodarone's effect on cell mobility, a pivotal step in cancer progression. In this, cells were seeded in the upper compartment containing 1% FCS and were allowed to migrate to the underside of this higher compartment which contained 10% FCS. After 18 h, migrated cells were counted as presented in Fig. 1B. Doses of 10 and 50 µM of Amiodarone significantly inhibited cell mobility, with an ∽81% and ∽92% reduction, respectively.

### Amiodarone increases susceptibility of U-87 MG cancer cells to anoikis

**Figure 2:**
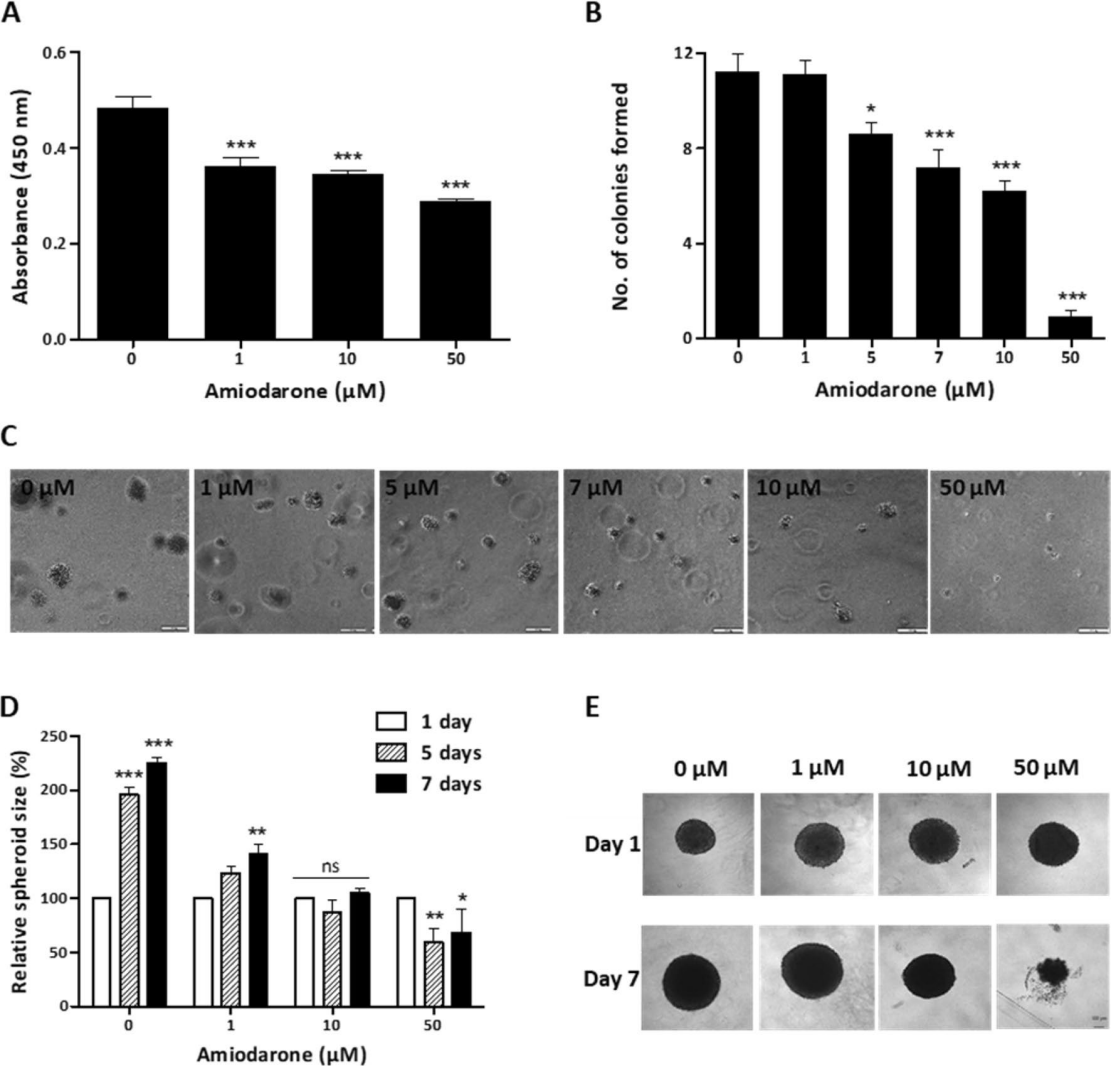
Amiodarone affects U-87 MG cell function in 3D. (**A**) Anoikis assay with U87-MG cells, using p-HEMA coated plates, incubated with ranging concentrations of Amiodarone (0, 1, 10 and 50 μM) for 72 h. Cell viability measured using WST8 assay, *n* = 6. ***p < 0.001, compared with non-treated control cells. (**B**, **C**) Soft agar colony formation assay of U-87 MG cells cultured for 15 days under ranging concentrations of Amiodarone (0, 1, 5, 7, 10, and 50 μM). Colonies visualized using inverted microscope on day 15. Images processed, analyzed, then average number of colonies per well using ImageJ calculated. *n* = 3. *p < 0.05, ***p < 0.001, compared with non-treated control cells. Scale bar: 100 µm. (**D**, **E**) Spheroid assembly and growth assay of U-87 MG cell spheroids. Relative spheroid size grown in rounded-bottom 96-well plate after 1, 5 and 7 days under ranging concentrations of Amiodarone (0, 1, 10 and 50 μM). Calculated using MATLAB code analysis (as shown in Fig. 2–figure supplement 1). Further normalization done in the plots—spheroid growth calculated as the ratio between the current size and the initial size, scaling the initial size values of day 1 as 100%, *n* = 3. *p < 0.05, **p < 0.01, ***p < 0.001, ns, not significant. Results are presented as mean ± SEM. Scale bar: 100 µm. Figure supplement 1. Amiodarone’s effect on relative spheroid size.

We evaluated Amiodarone’s effect on non-adherent cell survival, since cell survival in the blood circulation is a crucial factor in metastatic development^15^. In this assay, we examined the ability of detached U-87 MG cells treated with Amiodarone to withstand apoptosis for 72 h on a non-adherent surface generated by poly(2-hydroxyethyl methacrylate) (pHEMA). Figure 2A shows that control cells had higher survival rates than treated cells: 1 µM and 10 µM significantly reduced cell viability by 34% and 38%, respectively, compared with untreated cells. A marked decrease of 48% in cell viability was detected with 50 µM compared with untreated cells.

### Amiodarone reduces colony formation of U-87 MG cells

There is a correlation between the malignant behavior of tumors and their ability to form colonies in different types of matrixes^16^. In order to investigate whether Amiodarone affects U-87 MG cells’ ability to form colonies, we performed a soft agar colony formation assay over 15 days. Figure 2B,C show Amiodarone’s dose-dependent effect on the cells’ ability to establish colonies. Cells treated with 5, 7 and 10 µM Amiodarone presented a significant reduction in the number of colonies formed, 23%, 36% and 45%, respectively. Notably, 50 µM of Amiodarone exhibited almost a complete reduction of 92% in the number of colonies formed.

### Amiodarone inhibits U-87 MG spheroid growth

Spatial cell–cell interaction and proliferation of U-87 MG cells can be monitored using a three-dimensional (3D) spheroid assay which consists of an assembly step and cell growth. Relative spheroid size grown in rounded-bottom 96-well plates under varying concentrations of Amiodarone (0, 1, 10 and 50 µM) was calculated using MATLAB code analysis, as previously shown^17^, after 1, 5 and 7 days, as mentioned in the Materials and Methods section (Fig. 2–figure supplement 1). Cells under all conditions assembled into spheroids within 24 h of cell seeding (Fig. 2D, E). All control and 1 µM Amiodarone-treated spheroids proliferated and increased in size over time. The control spheroids more than doubled their size after 5–7 days, whereas spheroids treated with 1 µM increased by only ∽40% up to day 7. Spheroids treated with 10 µM Amiodarone preserved their size and did not show further growth. However, 50 µM Amiodarone reduced spheroid size on days 5–7 by ∽30–40%.

### Amiodarone exhibits anti-angiogenic activity in vitro in HUVECs

**Figure 3:**
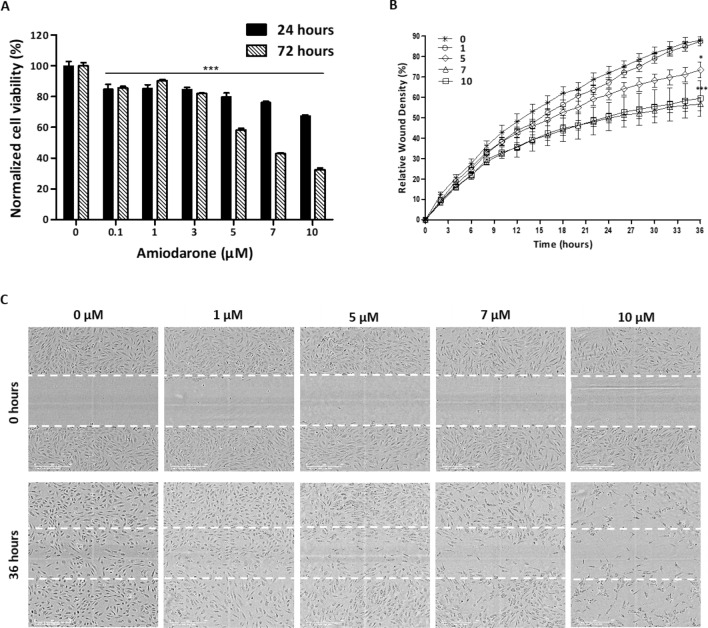
Amiodarone affects HUVEC function. (**A**) Viability assay of HUVECs, using WST8, after 24 and 72 h of incubation with ranging concentrations of Amiodarone (0, 0.1, 1, 3, 5, 7, and 10 μM), investigating cytotoxic and proliferation-rate effects, respectively, n = 8. ***p < 0.001, compared with untreated control cells with the same incubation time. (**B**, **C**) Scratch assay of HUVECs after 36 h of incubation using the IncuCyte S3 Live-Cell Analysis System, with ranging concentrations of Amiodarone (0, 1, 5, 7, and 10 μM), n = 8. Scale bar: 400 µm. *p < 0.05, ***p < 0.001, compared with non-treated control cells. Results are presented as mean ± SEM. Figure supplement 1. Amiodarone reduces cell viability of endothelial, fibroblast and melanoma cells.

Since angiogenesis is a prominent feature of many tumors, having a strong impact on tumor growth and progression^18–20^, the functionallity of endothelial cells was investigated. Human umbilical vein endothelial cell (HUVEC) viability, mobility and functionality were assessed under Amiodarone treatment. HUVEC cytotoxicity and proliferation assays were measured after 24 and 72 h of drug incubation, respectively. Amiodarone induced cytotoxic effects in all the concentrations used (0.1 – 10 μM), while 10 μM led to a 32% reduction in cell viability. Even the lowest concentration used, 0.1 µM, significantly reduced HUVEC proliferation (Fig. 3A). 0.1, 1 and 3 µM of the drug reduced cell proliferation by 10–18%. A significant dose-dependent effect was observed with higher concentrations of Amiodarone; 5, 7 and 10 µM induced a reduction of 42%, 57% and 67%, respectively. These results, combined with the MTT assay results of HUVECs, fibroblast and melanoma cells (Fig. 3–figure supplement 1), imply that HUVECs are more susceptible to Amiodarone’s cytotoxic and inhibiting action as compared with all the above-mentioned cells.

The drug’s effect on HUVEC mobility was measured in the scratch assay. After 36 h of exposure to the drug, 1 µM Amiodarone-treated cells nearly populated the entire scratch wound area (relative wound density ∽90%), much like the untreated cells shown in Fig. 3B,C. The cells’ migration decreased by ∽15% with 5 µM and by ∽30% with 7 and 10 μM.

**Figure 4:**
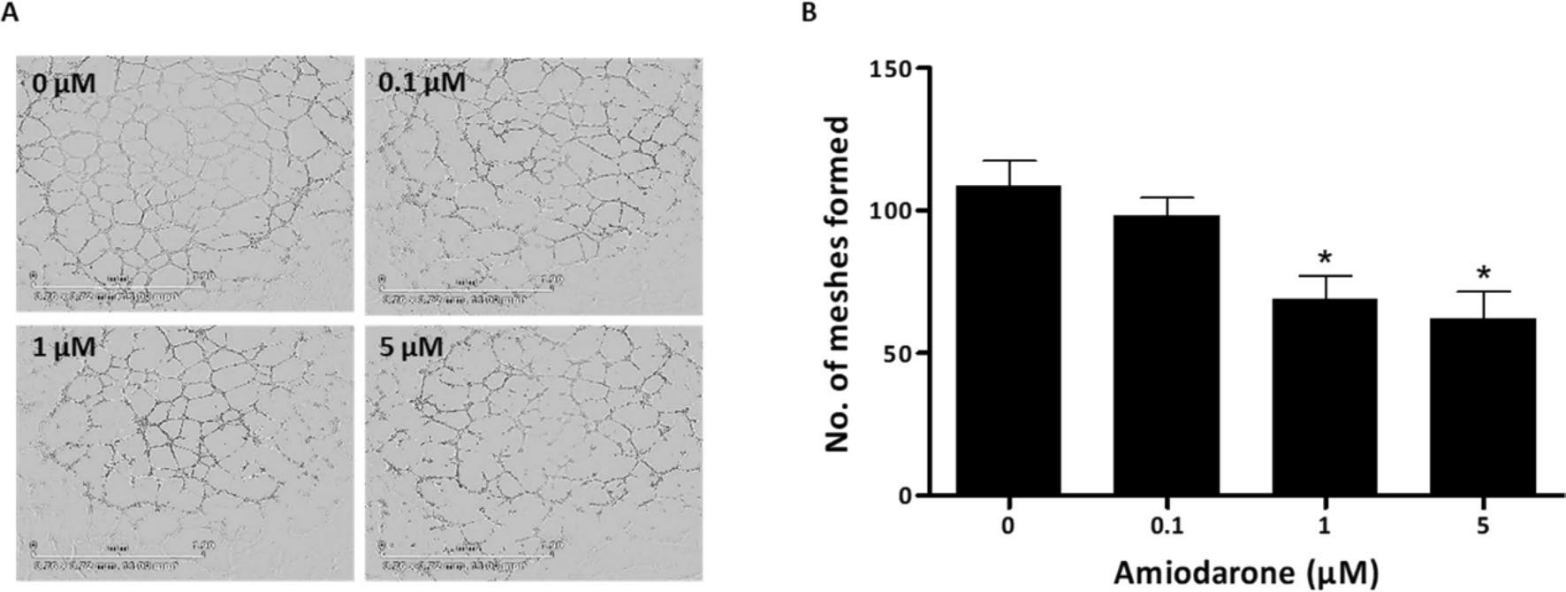
Amiodarone affects HUVEC function in 3D. (**A**, **B**) Angiogenesis tube formation assay of HUVECs seeded on Matrigel after 6 h of incubation, using IncuCyte S3 Live-Cell Analysis System with ranging concentrations of Amiodarone (0, 0.1, 1 and 5 μM). Statistical analysis of tube formation hallmark (number of meshes formed) and images processed using ImageJ software; representative images are presented, n = 4. Scale bar: 1.9 mm. *p < 0.05, compared with non-treated control cells. Results are presented as mean ± SEM.

The tube formation assay predicts the ability of endothelial cells to form stable tubes/branches by mimicking in vivo angiogenesis^21^. In this assay, HUVECs treated with 0.1 µM Amiodarone showed no significant effect; however, cells treated with drug concentrations of 1 and 5 µM reduced by ∽40% the number of meshes and branches formed compared with untreated cells Fig. 4A,B.

### Systemic amiodarone treatment has an anti-angiogenic effect in the mouse corneal neovascularization model

**Figure 5:**
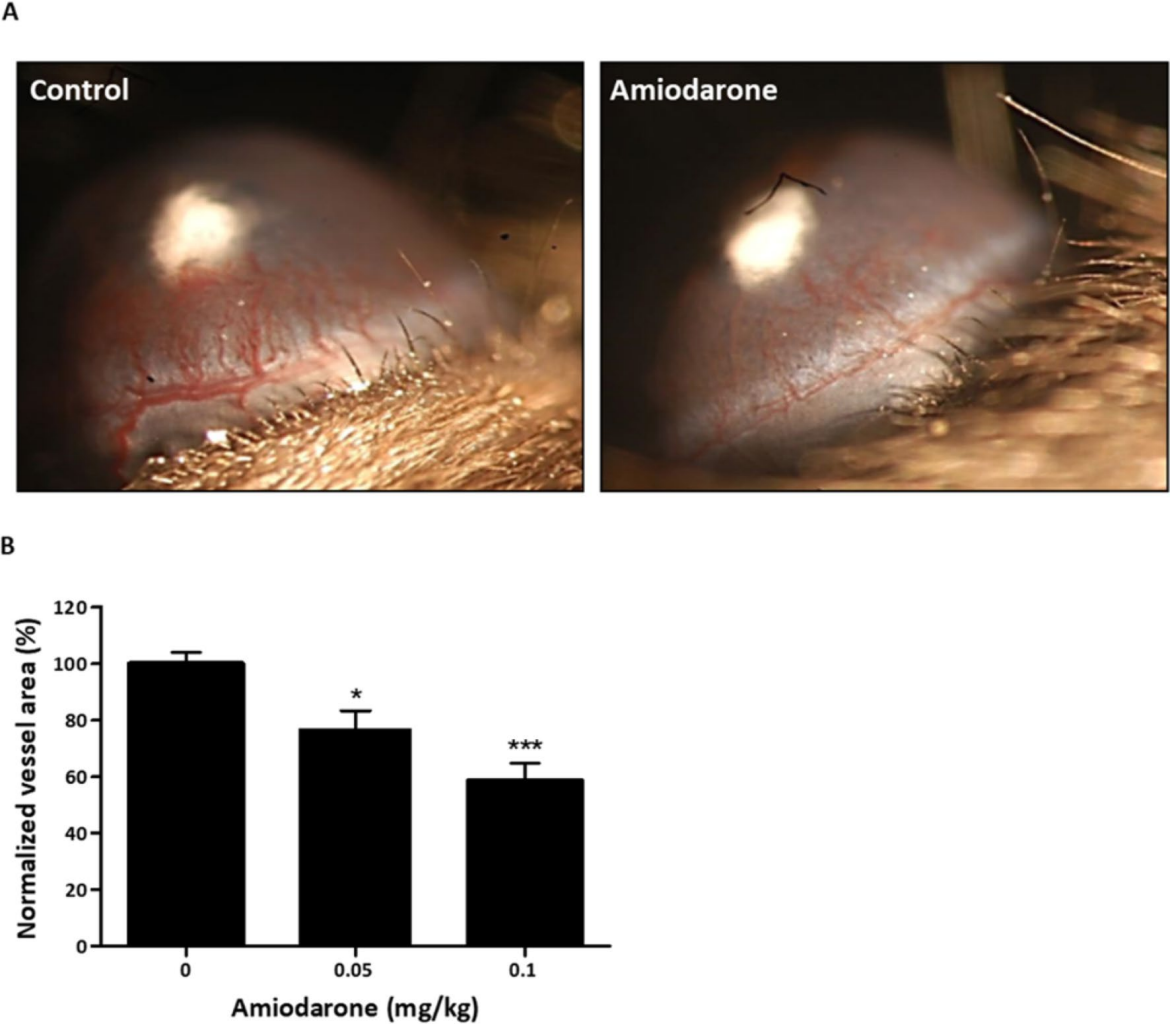
Amiodarone inhibits corneal neovascularization after I.P. treatment. Amiodarone’s anti-angiogenic activity evaluated following systemic administration in the mouse corneal micropocket assay using bFGF-induced neovascularization. C57BL/6 mice were injected I.P. with Amiodarone 0.05 mg/kg or 0.1 mg/kg q.d. for 5 days. (**A**) Representative images taken from treated (0.1 mg/kg q.d.) and untreated mice on day 5. bFGF pellets detected as a white spot in the center of the cornea. Blood vessels growing from limbal periphery are reduced in the Amiodarone-treated group compared with the untreated group. (**B**) Normalization of vessel area (%) for untreated and Amiodarone treated mice (0.05 mg/kg and 0.1 mg/kg q.d.). Vessel area was calculated using the following formula: [Clock hours × Vessel length (mm) × π × 0.2 mm]. Graphs indicate significant inhibition of vessel formation after 5 days of treatment. Objective lens 400 × , (in each group n = 10). *p < 0.05, ***p < 0.001, compared with non-treated control mice. Results are presented as mean ± SEM.

The mouse corneal micropocket assay using basic-fibroblast growth factor (bFGF)-induced neovascularization was utilized to evaluate the direct anti-angiogenic activity of Amiodarone following systemic administration in C57BL/6 mice. Amiodarone was administered intraperitoneally (I.P.), 0.05 mg/kg or 0.1 mg/kg q.d., over a course of 5 days. These treatments resulted in a reduction of 22% or 41%, respectively, in the corneal vessel area compared with the control (Fig. 5A,B) with no evidence of side effects. These results indicate a significant inhibition of vessel formation after 5 days of drug treatment.

### Amiodarone decreases angiogenesis in the matrigel plug assay in vivo

**Figure 6:**
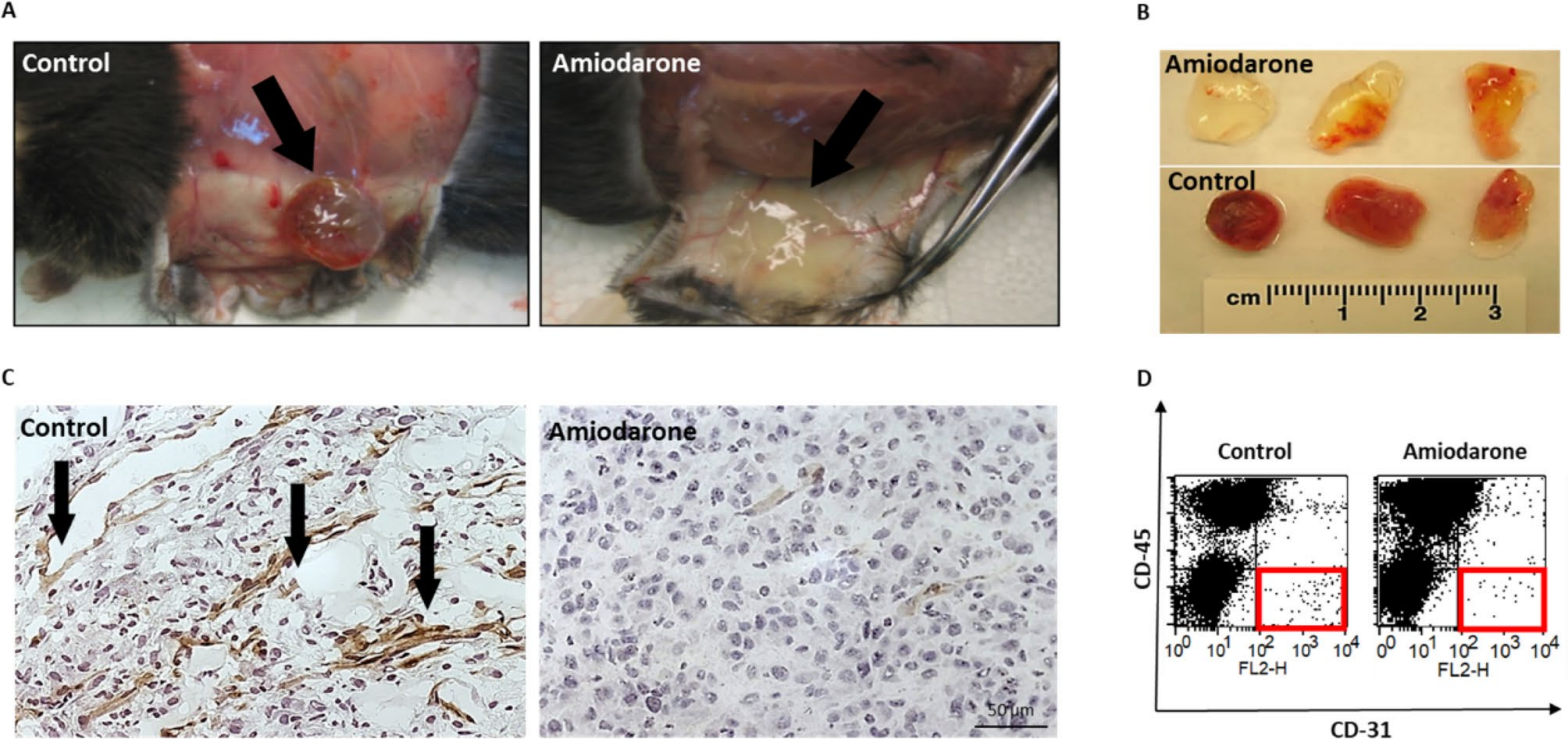
Amiodarone inhibits angiogenesis in Matrigel plugs. Matrigel containing VEGF and bFGF (control) or Matrigel containing VEGF and bFGF mixed with Amiodarone 0.05 mg/plug was injected S.C. into C57BL/6 mice to determine the drug’s effect on angiogenesis. (**A**, **B**) Representative plugs removed from Amiodarone-treated and untreated mice after 8 days. Untreated mice presented bloody plugs surrounded by massive blood vessels, compared with Amiodarone-treated mice which had poor vasculature. (**C**) Representative histologic sections of untreated plug (left) and Amiodarone-treated plug (right) of mice post anti-CD31 antibody (DAB-brown) and Gill’s Hematoxylin for nuclei (blue) staining. Arrows point to large vessels with open lumens. Scale bar: 50 µm. (**D**) Quantification of infiltrating endothelial cells in Matrigel plugs employing the flow cytometry assay. FACS dot plots of endothelial cells (CD31 + /CD45, marked in red squares) from single-cell suspension originated from untreated (left) and treated (right) Matrigel plugs. Data are presented as percent of specific cell population out of the total cell population (*p* < 0.05). Infiltrating endothelial cells were reduced by 60% in the Amiodarone-treated mice group compared with the non-treated control group. n = 3. Objective lens 400x.

Amiodarone was locally administered in a quantitative mouse Matrigel angiogenesis assay to investigate its ability to inhibit angiogenesis. Matrigel containing vascular endothelial growth factor (VEGF) and bFGF mixed with Amiodarone 0.05 mg/plug was injected subcutaneously (S.C.) into C57BL/6 mice. On day 8, immunohistochemical staining of blood vessels was performed using anti-CD31 antibodies, enabling visual evaluation of the vasculature in the Matrigel plugs (Fig. 6C). The Matrigel plugs mixed with Amiodarone showed significantly reduced blood vessel formation, as can be visually assessed in Fig. 6A-C. Untreated mice presented bloody plugs surrounded by massive blood vessels with open lumen structures, suggesting functional vessels, compared with Amiodarone-treated mice that showed poor vasculature. Quantification of the infiltrated endothelial cells in Matrigel plugs was accomplished in a single-cell suspension by Fluorescence-Activated Cell Sorting (FACS) (Fig. 6D). Infiltrating endothelial cells were substantially reduced by 60% in the Amiodarone-treated mice group compared with the control group.

### Amiodarone suppresses tumor growth and vasculature Of GBM xenografts

**Figure 7:**
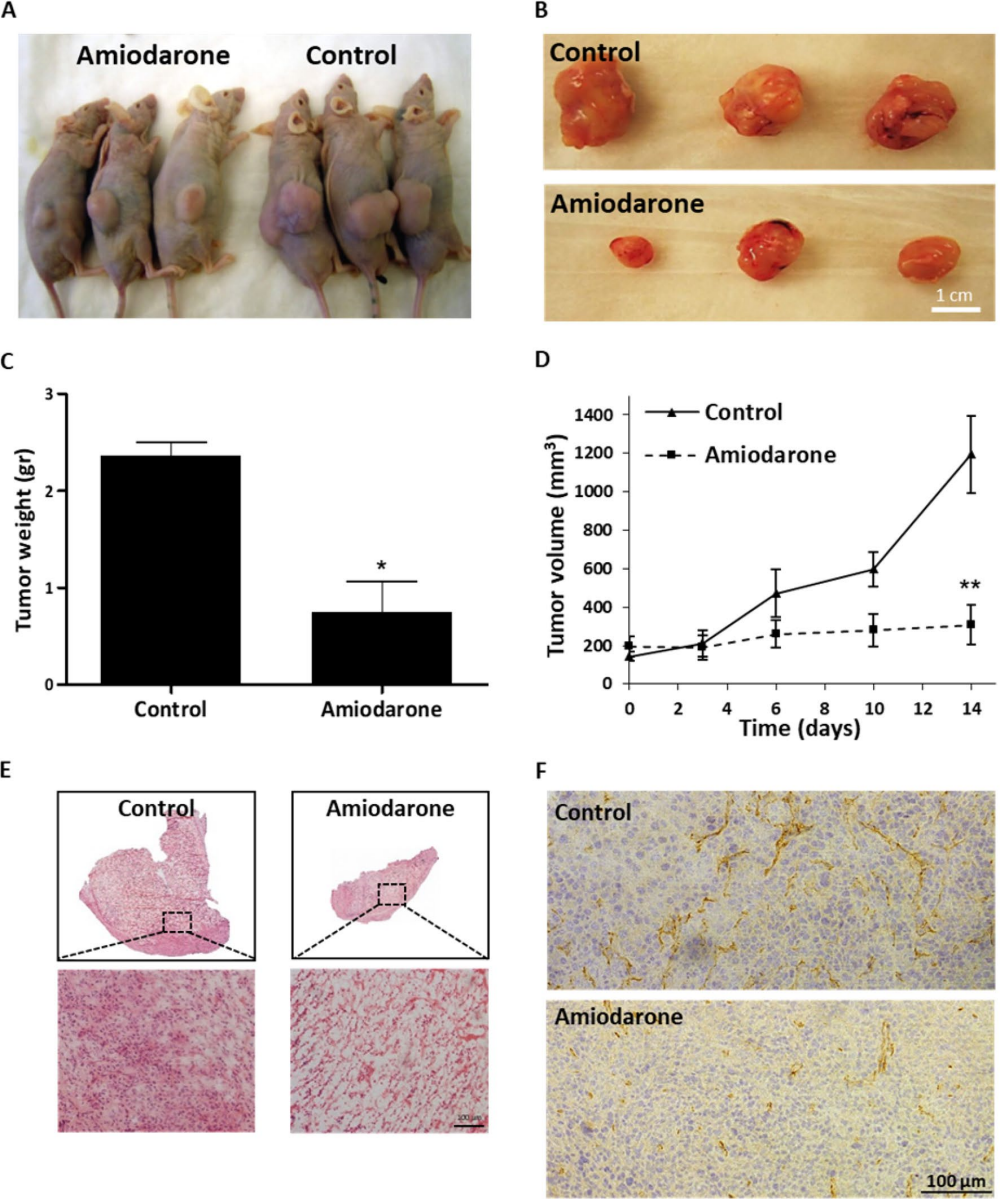
Amiodarone inhibits GBM tumor xenograft growth. Amiodarone’s anti-tumor activity following systemic administration (I.P.) evaluated in a S.C. tumor model. Foxn1 nu mice were inoculated S.C. with 5 × 106 U-87 MG cells. When tumors reached an average volume of ~ 200 mm^3^, mice were divided into two groups of treated (Amiodarone 0.1 mg/kg I.P. injection q.d.) and untreated (given saline) mice over a course of 14 days. (**A**, **B**) Tumors were surgically resected and collected when an average volume of ~ 1000 mm^3^ (day 14) was reached. (**C**, **D**) Weight and volume of tumors from treated and untreated mice were measured. Tumor volume was calculated using the standard equation (length × width^2^ × 0.52). n = 3–5. *p < 0.05, **p < 0.01, compared with non-treated control mice. Results are presented as mean ± SEM. (**E**) Representative histologic sections of untreated tumor (left) and Amiodarone-treated tumor (right) of mice post-H&E staining. Scale bar: 100 µm. (**F**) Representative images of tumor sections stained with anti-CD31 (DAB-brown) and Gill’s Hematoxylin for nuclei staining (blue). Untreated tumor (top) and Amiodarone-treated tumor (bottom). Scale bar: 100 µm. Objective lens 10 × . The data of harvesting livers, spleens and lungs of control mice and Amiodarone-treated mice on day 14 are not shown here but are discussed in the Results section and in Fig. 7–figure supplement 1. The data of Amiodarone 0.05 mg/kg I.P. injection q.d. treatments are discussed in the Results section and shown in Fig. 7–figure supplement 2. Figure supplement 1. Effect of Amiodarone in GBM tumor xenograft. Figure supplement 2. Amiodarone reduces GBM tumor xenograft volume.

For the in vivo anti-tumor activity of Amiodarone, U-87 MG cells were evaluated in S.C. injected Foxn1 nu mice. When the tumors reached an average volume of ∽200 mm^3^, the mice were divided into two groups of treated (Amiodarone 0.1 mg/kg q.d. I.P. injection) and untreated mice. After the experiment was terminated, when an average volume of ∽1 000 mm^3^ was reached (on day 14), the tumors were surgically resected and collected for histological examination. Significant differences in tumor size and weight between the treated and untreated groups were observed, with average tumor weights of 0.75 gr or 2.36 gr (∽threefold reduction) and average tumor volumes of 308 mm^3^ or 1106.25 mm^3^ (> 72% reduction), respectively (Fig. 7A-D). Histological Hematoxylin/Eosin (H&E) staining showed that in addition to reduction in volume, the treated tissues exhibited a more porous and defective structure compared with untreated tumors (Fig. 7E). This is possibly due to increased necrosis correlating with our in vitro flow cytometry analyses of U-87 MG Annexin-V-APC and PI cell staining (Fig. 1–figure supplement 1). Immunohistochemical staining of microvessels with anti-CD31 antibodies presented notably lower vascularization compared with untreated tumors (Fig. 7F). Gross pathology revealed that spleens retrieved from the control group displayed significant splenomegaly compared with the Amiodarone-treated mice (Fig. 7–figure supplement 1). With the 0.1 mg/kg q.d. I.P. injection treatment, there were some indications of toxicity in the form of stomach swelling, abnormal liver, and weight loss of up to 14%. Two out of the five mice died on day 10 and 14. From the control group one mouse died on day 14. Reducing the dose to a 0.05 mg/kg q.d. I.P. showed good safety without any significant weight loss while still maintaining effective activity, reducing tumor volume by 40% compared with the control mice on day 24 (Fig. 7–figure supplement 2).

## Discussion

Amiodarone, a widely administered anti-arrhythmic drug^4^, was shown by several pre-clinical studies to reverse multidrug resistance and sensitize several types of cancers to chemotherapy. Pre-clinical studies demonstrated its anti-cancer effects as a single therapeutic or in combination with other anti-cancer drugs on different cancers, including prostate, hepatocellular and breast carcinomas^6–9,22–24^. Based on this, we opted to investigate its anti-cancer effects on a human GBM cell line, U-87 MG, a common cancer model used to form aggressive and angiogenic tumor xenografts^13^. Finding drugs with dual effects that can target both cancer cells and angiogenesis is of high value in combating highly angiogenic tumors. We hypothesized that, in addition to Amiodarone's previously demonstrated anti-cancer effects, it also exhibits prominent anti-angiogenic activity, consequently offering a possible new approach to treating highly vascularized tumors.

The direct anti-cancer effects of Amiodarone on U-87 MG cells were demonstrated in a wide variety of in vitro assays. Its most prominent effect was shown to be on cell mobility as demonstrated in the migration assay, where 10 µM of Amiodarone led to a more than 80% inhibition in the number of migrated cells. Proliferation was also reduced with exposure to Amiodarone, but in higher doses (~ 60% inhibition was obtained with 50 µM).

The observed effect of the drug on cell viability may be related to the induction of cell death as previously shown. Kim et al. demonstrated that Amiodarone induced apoptosis in U-87 MG cells in a dose-dependent manner, and this effect was synergistically enhanced on various malignant glioma cells when combined with tumor necrosis factor (TNF)-related apoptosis-inducing ligand. The associated effect was shown to be mediated by induction of the unfolded protein response (UPR) and an increase of intracellular Ca^2+^ levels, which induced cell death^23^.

To further investigate Amiodarone's ability to impede the metastatic potential of cancer cells, we measured its effect on the survival of cells in circulation. Anoikis is a programmed cell death activated when cells lose their contact with the extracellular matrix (ECM) occurring during their invasion into the blood circulation. Cancer cells that succeed in evading anoikis may then colonize in distant organs and begin the metastatic process^15^. We show that Amiodarone in the range of 1–50 µM, reduced the survival of U-87 MG cells on a non-adherent surface, by ~ 30–50% (Fig. 2A).

The colonization potential of cancer cells involves an anchorage-independent process requiring cell–cell interactions and proliferation^25^. In our experiments, Amiodarone reduced colony formation and significantly inhibited U-87 MG cells’ ability to form aggregates in soft agar in a dose-dependent manner, > 90% inhibition. Recently published data by Chang et al. showed a similar trend of Amiodarone's effect on cervical carcinoma cell colonization, suggesting an associated increase in truncated serine and arginine-rich splicing factor 3 (SRSF3), and reduction of RNA (miR)-224. Amiodarone's suppressive effects on the SRSF3 promoter activity were further verified in GBM8401 and U118MG glioma cell lines^6^.

Similar to colony formation that requires 3D cell interactions, the multicellular spheroid model provides a physiologically relevant model in an ECM-free condition. Unlike monolayer culture, spheroids enable spatial cell–cell interactions and the generation of tissue-like features^17,26^. In the spheroid model, Amiodarone was found to significantly suppress spheroid growth, while 50 µM also induced a notable shrinkage in size. Similar results were reported for patient-derived glioblastoma stem-like cultures showing induction of apoptosis and decreased invasion of tumor neurospheres in association with change in cell cycle and survival gene expression^27^. These results are in agreement with other reports demonstrating that Amiodarone inhibited cancer cell migration in vitro, and inhibited invasion in a Matrigel assay of human breast cancer cells MDA-MB-231, as well as other murine tumor cells, B16OVA melanoma, JC and 4 T-1 breast cancer^24^. Our results point to Amiodarone's potential for reducing tumorigenesis and invasiveness, as demonstrated by the confirmed reduction in motility, invasiveness, proliferation, adherence-free survival and colonization.

It is well established that the tumor microenvironment and, more specifically, angiogenesis, have an immense impact on tumor growth^20,28^. Amiodarone's direct effect on endothelial cell function was measured in many in vitro assays using HUVECs. We found that HUVECs were more sensitive to Amiodarone compared with U-87 MG cells, NIH 3T3 fibroblasts and B16-F10 melanoma cells with respect to viability (Fig. 1A, Fig. 3A and Fig. 3–figure supplement 1). Our observations are in agreement with previous studies indicating that Amiodarone may have selective activity, showing relatively nontoxic effects with normal astrocytes while inducing apoptosis in glioma cells^23,27^. In concentrations ranging from 0.1 to 10 µM, Amiodarone significantly reduced cell proliferation (up to ~ 70%), cell mobility (∽30%) and formation of tubes/branches (∽40%) (Fig. 3, 4). Considering that angiogenesis involves a series of events, including endothelial cell migration, proliferation and tubular formation^18^, our in vitro results imply that Amiodarone may have a clinically relevant anti-angiogenic effect. Moreover, these in vitro results are in line with our in vivo results of the angiogenesis model — the Matrigel plug assay^29^. This model provides a direct assessment for endothelial cell recruitment and their morphogenesis into the blood vessel network. In this animal study, Amiodarone that was mixed into Matrigel plugs (0.05 mg/plug), as a mode of local treatment, extensively reduced the vascularization of the plugs over the course of 8 days. A similar trend was confirmed in the mouse corneal micropocket angiogenesis assay, where Amiodarone, administered systemically over a course of 5 days, induced up to a 40% reduction of vessel formation with no evidence of side effects.

GBM tumor xenografts provide an overall assessment of tumor progression, including both direct processes of cancer cells and indirect anti-cancer tumor microenvironment processes. Amiodarone at the dose of 0.1 mg/kg q.d. I.P., over a course of 14 days, showed over a 70% reduction in tumor volume and weight. Immunohistological staining confirmed an associated reduction in angiogenesis, where untreated tumors showed rich microvessel networks compared with treated mice. The drug's underlying mechanism of action is yet unclear and involves multiple possible target sites, but taken together, our results suggest that Amiodarone possesses anti-angiogenic attributes along with its anti-tumor activity — a dual effect.

One of the key issues with Amiodarone is its adverse side effects when used to treat cardiac dysrhythmias. These include: dangerous polymorphic ventricular arrhythmia due to its blocking potassium channels^30^; dermatologic changes, including photosensitivity and blue-gray skin discoloration^31^; and thyrotoxicosis due to its similarity to thyroxine^11,32^. But the drug's most serious side effect is pulmonary toxicity that can lead to pulmonary fibrosis and death^33^. These clinical symptoms may last months after cessation due to a long biological half-life^12^.

Given this, our study highlights the fact that the anti-cancer activity in the GBM xenograft model can be obtained with a low dose of Amiodarone compared with the human equivalent dose that is currently prescribed for cardiovascular conditions. The dose of 0.1 mg/kg q.d. I.P. in mice showed some signs of toxicity. However, half of this dose was well tolerated over a course of 24 days, while still inducing a significant reduction in tumor growth. When the same doses of 0.1 mg/kg or 0.05 mg/kg q.d. I.P. were administered for 5 days in the mouse corneal micropocket assays, no side effects were evident, suggesting the possibility of fine tuning the dosage for chronic treatment. Since the pharmacokinetics of substances administered I.P. are similar to those of oral administration^34^, it can be concluded that our highest dose administered to mice is equivalent to 0.48 mg/day in a human adult, based on the FDA's guidelines and Reagan-Shaw's conversion formula^35,36^. This is a significantly lower dose than those used for treating antiarrhythmic diseases (typically between 100 to 400 mg/day^37^), which is between 200 and 800-fold lower. The positive outcomes using a low dose, combined with the drug’s dual effect, emphasizes Amiodarone’s great potential for treating highly vascularized tumors, either systemically or locally.

Taken together, our in vitro and in vivo results suggest that Amiodarone possesses both significant anti-cancer activity — demonstrated by reduction of tumorigenic processes directly related to tumor progression, such as invasion and colonization — and significant anti-angiogenic activity. We found that low dose Amiodarone induced tumor shrinkage in GBM xenografts and profoundly reduced blood vessel formation in all mouse models.

Repurposing Amiodarone for low dose systemic or local treatment, in light of its suggested multi-targeting mechanisms, may bypass the drug’s negative side effects and become a new standard for treating solid tumors, particularly ones that are largely dependent on angiogenesis.

## Materials and methods

### Compliance with ethical standards

Mice (C57BL/6 J and Foxn1 nu) were purchased from Jackson Laboratories (Bar Harbor, ME, USA). All protocols were approved by the Institutional Animal Care and Use Committee at Children’s Hospital Boston and were conducted in accordance with the Association for Research in Vision and Ophthalmology’s Statement for the Use of Animals in Ophthalmic and Vision Research.

### Cell culture

Human glioblastoma cell line U-87 MG, mouse fibroblast cell line NIH 3T3 and mouse melanoma cell line B16-F10 were purchased from ATCC (VA, USA). HUVECs were obtained from Lonza (Switzerland). All cells were characterized before use, mycoplasma free (using an EZ-PCR Mycoplasma Test Kit (Biological Industries)) and used for the experiments up to p15. All cells were kept in a humidified incubator at 37 °C with 5% CO_2_. For U-87 MG cells EMEM-conditioned medium (Life Technologies, MA, USA) was used and supplemented with 1% L-glutamine, 1% sodium pyruvate, 10% fetal calf serum (FCS) and penicillin/streptomycin. NIH 3T3 and B16-F10 cells were cultured in DMEM-conditioned medium (Life Technologies, MA, USA) supplemented with 10% FCS and penicillin/streptomycin. HUVECs were maintained in a specific medium supplemented with PeproGrow-MacroV kit (ENDO-BM & GS-MacroV, PeproTech) and penicillin/streptomycin, and were seeded in wells that were previously coated with gelatin 0.1% (Sigma-Aldrich).

### Cytotoxicity assay of U-87 MG, NIH 3T3 and B16-F10 cells and HUVECs

Each well of the 96-well culture plates (Thermo Fisher Scientific NuNclon Delta Surface, Denmark) was seeded with proper medium containing U-87 MG cells (8 000 cells/well) or HUVEC (6 000 cells/well) and incubated in 37 °C and 5% CO_2_ for 24 h. Amiodarone (INJ 50 mg/ml, Sanofi-Winthrop, France) was added to each well in varying concentrations (0, 1, 10, and 50 μM with U-87 MG cells, and 0, 0.1, 1, 3, 5, 7 and 10 μM with HUVECs). Stock solution of Amiodarone contains 2% Benzyl alcohol (further diluted), therefore, the proper concentration of benzyl alcohol was added to each treatment. The plate was incubated in 37 °C and 5% CO_2_ for another 24 h. After incubation, WST-8 reagent (Sigma-Aldrich, Japan) was added into each well for viability detection and incubated in 37 °C and 5% CO_2_ for 1 h. Absorbance was measured at 450 nm using a plate reader (Wallac 1420 VICTOR plate-reader, Perkin-Elmer Life Sciences, USA), *n* = 10. For the MTT assay, HUVECs, NIH 3T3 and B16-F10 cells were seeded in 96-well culture plates (5000 cells/well) with the proper medium and let to incubate in 37 °C and 5% CO_2_ for 24 h. Amiodarone was added to each well in varying concentrations (0, 0.01, 0.5, 1, 50, and 100 μM) after which the plates were left to incubate for another 24 h. After incubation, 3-(4,5-methylthiazol-2-yl)-2,5-diphenyltetrazolium bromide (MTT) reagent (Sigma-Aldrich, Japan) was added into each well for viability detection and incubated in 37 °C and 5% CO_2_ for 1 h. Absorbance was measured at 570 nm using a plate reader, *n* = 3. 300 000 U-87 MG cells/well were seeded in 6-well plates for the flow cytometry assay. Cells were incubated with ranging concentrations of Amiodarone (0, 10 and 50 µM) for 24 h. Following drug treatments, the samples were stained using the Dead Cell Apoptosis kit with Annexin-V-APC and PI, according to the manufacturer's instructions (BioLegend, USA). Samples were acquired using a LSRFortessa Flow Cytometer (BD Biosciences, USA) and analyses were conducted using FlowJo software (Tree Star, Ashland, OR, USA), *n* = 5.

### Proliferation assay with U-87 MG cells and HUVECs

U-87 MG cells (3 000 cells/well) and HUVEC (3 000 cells/well) were incubated for 24 h in their proper conditioned media. Amiodarone in varying concentrations (0, 1, 10, and 50 μM for U-87 MG cells, and 0, 0.1, 1, 3, 5, 7 and 10 μM for HUVECs) was added into all wells. Plates were incubated in 37 °C and 5% CO_2_ for another 72 h followed by viability detection using WST-8 reagent, *n* = 9.

### Transwell migration

U-87 MG cells were harvested and centrifuged for 5 min at room temperature (RT). Next, cells were suspended in media containing 1% FCS and counted. For this assay a 24-well cultured plate with 8 μm pore size polycarbonate membrane transwell inserts (Corning Incorporated, USA) was used. Proper medium containing 10% FCS was added to the lower compartments. All inserts were adjusted to allow the membrane to be uniformly submerged in the medium. Next, 1% FCS medium containing 70 000 U-87 MG cells was added to each of the inserts' upper compartments and incubated at 37 °C and 5% CO_2_ for 30 min. After incubation, 1% FCS medium containing different concentrations of Amiodarone (0, 1, 10 and 50 µM) was added to each of the inserts' upper compartments and then incubated at 37 °C and 5% CO_2_ for 18 h to allow cells to migrate toward the underside of the inserts' filter. After 18 h, cells were fixed, stained and an inverted microscope (OLYMPUS IX73, Japan) was used to detect and count cells on the lower side of the inserts' filter (5 different fields per well) and the average number of migrated cells was calculated, *n* = 6.

### Anoikis assay

A pHEMA (Sigma, USA) culture was used to generate Anoikis. A solution containing 20 mg/ml pHEMA in 95% ethanol was prepared and left on a stirrer at RT to dissolve. Once dissolved, the solution was pipetted into 6-well culture plates (Thermo Fisher Scientific NuNclon Delta Surface, Denmark). The plates were left in a sterile biological safety cabinet until the ethanol evaporated, and the pHEMA solidified and coated the wells evenly. Proper growth medium containing different concentrations of Amiodarone (0, 1, 10 and 50 µM) was placed in each plate. All plates were seeded with 50 000 U-87 MG cells/well and incubated for 72 h at 37 °C and 5% CO_2_. After incubation, cell viability was measured using WST-8. Absorbance was measured as previously mentioned, *n* = 6.

### Soft agar assay

Soft agar assay was performed with U-87 MG cells. Six-well culture plates were coated with a bottom layer of 2% agar (Invitrogen by Life Technologies) and left to solidify in RT. 0.6% agar containing 30 000 U-87 MG cells/well was added to all wells left to solidify in RT. Proper medium containing different concentrations of Amiodarone (0, 1, 5, 7, 10, and 50 μM) was added to all wells. Plates were incubated at 37 °C and 5% CO_2_. Medium with Amiodarone treatment was refreshed every 5 days. On day 15, colonies were visualized using an inverted microscope (Nikon ECLIPSE TS100). Colonies were fixed, stained and counted (5 different fields per well). Images were processed, and the average number of colonies per well was analyzed and calculated using ImageJ, *n* = 3.

### Spheroid growth assay

15 000 U-87 MG cells/well were seeded in round-bottom 96-well plates (Lipidure, NY) coated with 2-methacryloyloxyethyl phosphorylcholine (MPC) and treated with varying concentrations of Amiodarone (0, 1, 10 and 50 µM). The plates were incubated at 37 °C and 5% CO_2,_ and images were taken 24 h, 5 and 7 days later using an inverted microscope, as mentioned before. MATLAB code was used to generate the calculated area of the different spheroids, as detailed in the spheroids' image analysis section below, *n* = 3.

Spheroid images were cropped to the exact same dimension so that unnecessary background (including pixels of the scale bar) were excluded. Using a MATLAB code, binary B&W images were then formed, in which live spheroids were distinguished from the background Fig. 2–figure supplement 1). The number of pixels was counted in each spheroid. Since individual pictures may be of different resolutions, the total number of pixels in the picture was counted as well and the scaled size of the spheroid was calculated as the ratio between spheroid pixels and the total number of pixels. In some cases, where many dead cells surrounded the live spheroid, the background was cleaned using Photoshop before the B&W image was constructed. In all cases, the scaled spheroids' size was calculated by the ratio between the number of spheroid pixels and the total number of pixels in the cropped image. A further normalization was done in the plots — the spheroid growth was calculated as the ratio between the current size and the initial size, scaling the initial size values of day 1 as 100%.

### Scratch wound migration assay

7 000 HUVECs/well were seeded in an IncuCyte ImageLock 96-well Plate (Sartorius, USA) and incubated in 37 °C and 5% CO_2_ for 24 h. Varying concentrations of Amiodarone (0, 0.1, 1, 3, 5 and 10 μM) were added to each well and a scratch wound was made in each well using the IncuCyte 96-well WoundMaker Tool (ESSEN, BioScience). The plate was placed in an incubator containing the IncuCyte S3 Live-Cell Analysis System (ESSEN, BioScience) for 36 h. Real time automated images were taken every 2 h and cell migration analysis was preformed using the IncuCyte Scratch Wound Cell Migration Software Module. Final images were processed using ImageJ, *n* = 9.

### Tube formation assay

An endothelial tube formation assay was performed with HUVECs. The wells' bottoms were coated with Matrigel Matrix (Corning) 50 μl/well and left to polymerize for 1 h at RT. This was followed by adding 200 μl of Amiodarone in varying concentrations (0, 0.1, 1 and 5 μM) combined with 20 000 cells/well to the Matrigel-coated 96-well plate. The plate was placed in an incubator containing the IncuCyte S3 Live-Cell Analysis System for 6 h. Real time automated images were taken each hour. Tube formation analysis (calculation of the number of meshes formed) and image processing were done using ImageJ, *n* = 4.

### Corneal micropocket assay

The corneal micropocket assay was carried out as previously detailed^38^ in C57BL/6 J mice. Micropockets in the mouse cornea were surgically created and pellets containing 80 ng of carrier-free recombinant human bFGF (R&D Systems, Minneapolis, MN) were implanted into them. Amiodarone was administered I.P., 0.1 mg/kg/d or 0.05 mg/kg/d q.d. over a course of 5 days. After 5 days, the vascular growth area was measured using a slit lamp and photos of mouse eyes were taken. The area of neovascularization was calculated as vessel area by the product of vessel length measured from the limbus and clock hours around the cornea, using the following equation: vessel area (mm^2^) = [clock hours × vessel length (mm) × π × 0.2 mm], *n* = 10.

### In vivo matrigel plug angiogenesis assay

C57BL/6 J mice were injected S.C. with growth-factor-reduced Matrigel Matrix (BD Biosciences) mixed with recombinant human VEGF (10 ng/ml) and bFGF (10 ng/ml). Matrigel plugs in the treated groups were additionally mixed with Amiodarone 0.05 mg/plug. After 8 days, Matrigel plugs were removed, embedded in optimal cutting temperature compound (OCT) medium (OCT, Tissue-Teck, USA) and immunohistochemistry was performed using a Vectastain Elite ABC kit (Vector Laboratories) followed by anti-CD31 (BD Biosciences) reaction for micro-vessel staining. Detection was carried out using a 3,3′-Diaminobenzidine chromogen (DAB), resulting in positive brown staining, in addition to using Gill’s Hematoxylin for nuclei staining. Quantification of the endothelial cells in Matrigel plugs was peformed by Flow cytometry using FACSCalibur and CellQuest software (BD Biosciences, San Jose, CA, USA) following enzymatic digestion of Matrigel plugs, as described elsewhere^39^. Immunostaining was performed for FACS analysis in the presence of rat anti-mouse CD45-APC and CD31 + PE (BD Biosciences). Endothelial cells were defined as CD31 + CD45- cells, *n* = 3.

### GBM tumor xenograft model

Foxn1 nu mice were inoculated S.C. with 5 × 10^6^ U-87 MG cells/mouse. Tumor growth was measured transcutaneously with a digital caliper every other day. Tumor volume was calculated using the standard equation: (length × width^2^ × 0.52). When tumors reached an average volume of ∽200 mm^3^, the mice were divided into groups of treated (Amiodarone 0.1 mg/kg or 0.05 mg/kg I.P. injection q.d.) and untreated (given saline) mice over a course of 14 or 24 days, respectively. Tumors were surgically resected and collected when an average volume of ∽1 000 mm^3^ was reached. Tumor weight and volume were measured, followed by embedment in OCT medium and prior to sectioning using a microtome-cryostat. Histological sections were stained using H&E or anti-CD31 (DAB) followed by Gill’s Hematoxylin, as previously mentioned, n = 3–5.

### Replications

The in vitro experiments were repeated independently at least twice, while the in vivo experiments were conducted two times, each with a different dose.

### Statistics

Statistical data was analyzed on GraphPad Prism 8 (www.graphpad.com, San Diego, CA). Studies containing two groups were assessed using the unpaired two-tailed Student’s *t*-test. Studies containing more than three groups were compared and analyzed using a one-way analysis of variance (ANOVA), and significant differences were detected using Tuckey's multiple comparison post-test. Spheroid radiuses were measured using MATLAB code (as detailed in the Materials and Methods section) and subsequently analyzed by two-way ANOVA. Differences were considered statistically significant for p < 0.05.

## Supplementary information

Supplementary Information
