# Supplementary material for: Low dose amiodarone reduces tumor growth and angiogenesis
Source: Sci Rep. 2020 Oct 22;10:18034. doi: 10.1038/s41598-020-75142-1 (PMC7582908; doi:10.1038/s41598-020-75142-1)
Supplement: Supplementary file 1 — Supplementary Information [file 41598_2020_75142_MOESM1_ESM.docx]

Low Dose Amiodarone Reduces Tumor Growth and Angiogenesis

Eliana Steinberg ^1^, Arnon Fluksman ^1^, Chalom Zemmour ^1^, Katerina Tischenko ^1^, Adi Karsch-Bluman ^1^, Yifat Brill-Karniely ^1^, Amy E Birsner ^2^, Robert J D'Amato ^2,3^ and Ofra Benny ^1,^*

^1^ The Institute for Drug Research, The School of Pharmacy, Faculty of Medicine, The Hebrew University of Jerusalem, Israel

^2^ Vascular Biology Program, Department of Surgery, Boston Children's Hospital, Harvard Medical School, Boston, Massachusetts, USA

^3^ Department of Ophthalmology, Boston Children's Hospital, Harvard Medical School, Boston, Massachusetts, USA

***** Correspondence: OfraB@ekmd.huji.ac.il (O.B.)

**Figure 1–figure supplement 1**

**
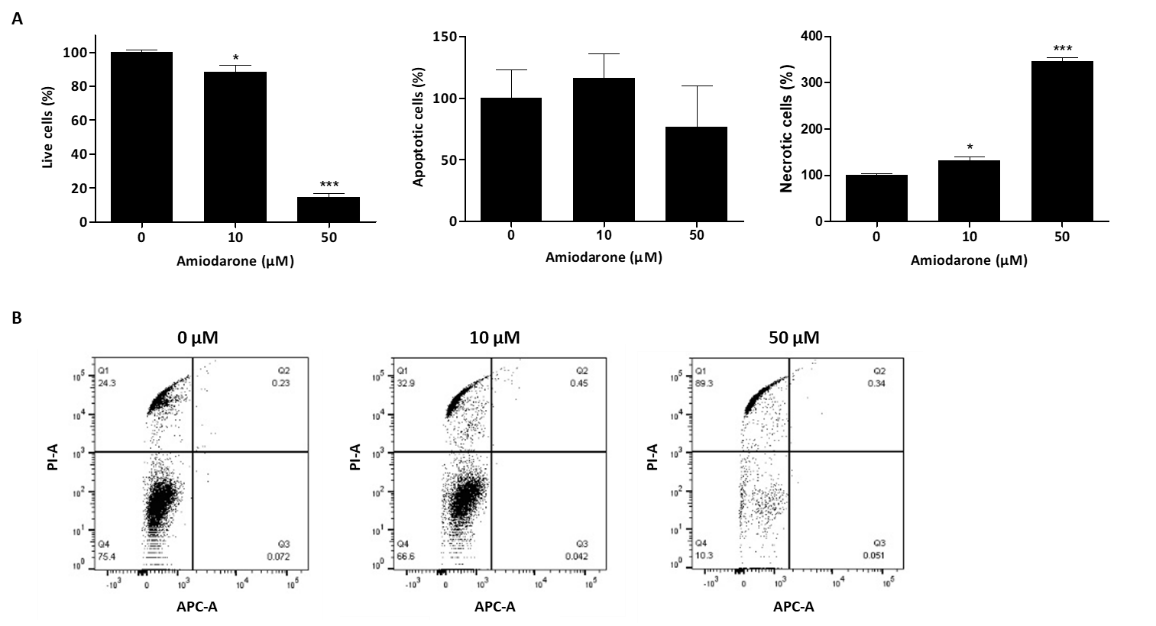
**

**Amiodarone's effect on U-87 MG cell viability.**Flow cytometry analyses of Annexin-V-APC and PI staining of U-87 MG cells incubated with ranging concentrations of Amiodarone (0, 10 and 50 µM) for 24 h. (**A**) Quantification of percentage of live, early apoptotic and necrotic U-87 MG cells. Data are presented as a percent of specific cell population normalized to non-treated control cells, scaling control cells as 100%. Late apoptotic cell population was not significant (data not shown). (**B**) Representative FACS dot plots of Annexin-V-APC and PI staining of U-87 MG cells from single-cell suspension. *n*=5. **p*<0.05, ****p*<0.001, compared with non-treated control cells. Results are presented as mean ± SEM.

**
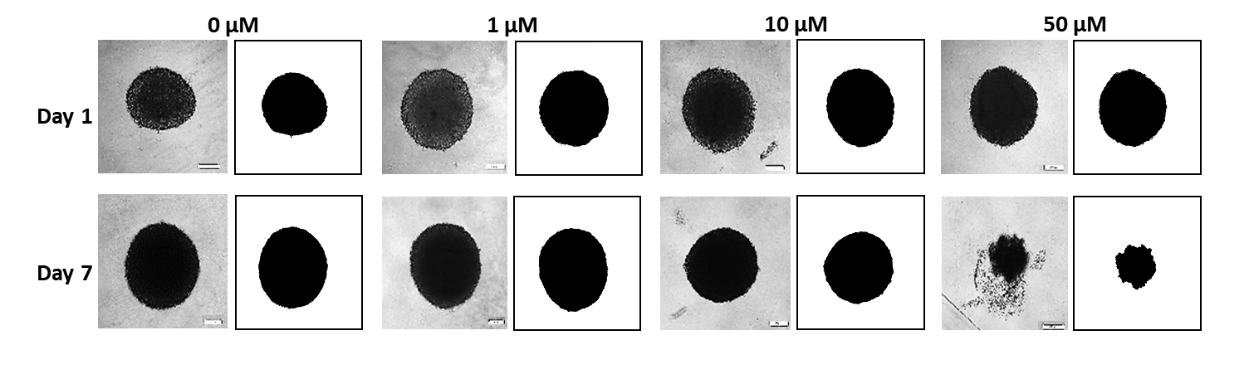
Figure 2–figure supplement 1**

**Amiodarone’s effect on relative spheroid size.**
Spheroids comprised of U-87 MG cells were grown in rounded-bottom 96-well plate. Representative images taken 1 and 7 days after treatment with ranging concentrations of Amiodarone. Calculated the relative spheroid size using MATLAB code analysis. Further normalization was done in the plots. *n*=3. Scale bar: 100 µm.

**Figure 3–figure supplement 1**

**
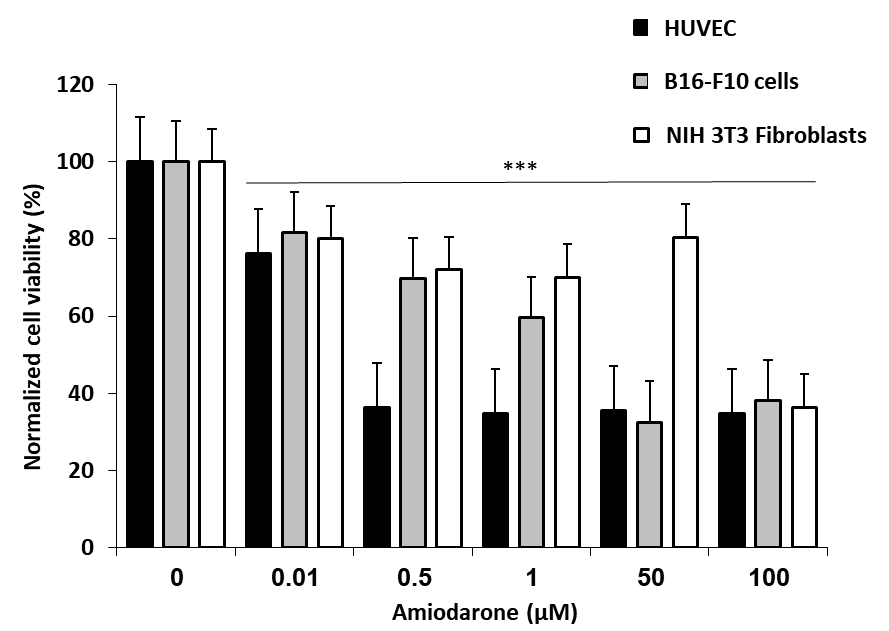
**

**Amiodarone reduces cell viability of endothelial, fibroblast and melanoma cells.**
Viability assay of HUVECs, B16-F10 melanoma and NIH 3T3 fibroblast cells using MTT after 24 h of incubation with ranging concentrations of Amiodarone (0, 0.01, 0.5, 1, 50 and 100 µM). HUVECs show a higher susceptibility to Amiodarone’s cytotoxic effects. n=3. ***p<0.001, compared with non-treated control cells. Results are presented as mean ± SEM.

**Figure 7–figure supplement 1**


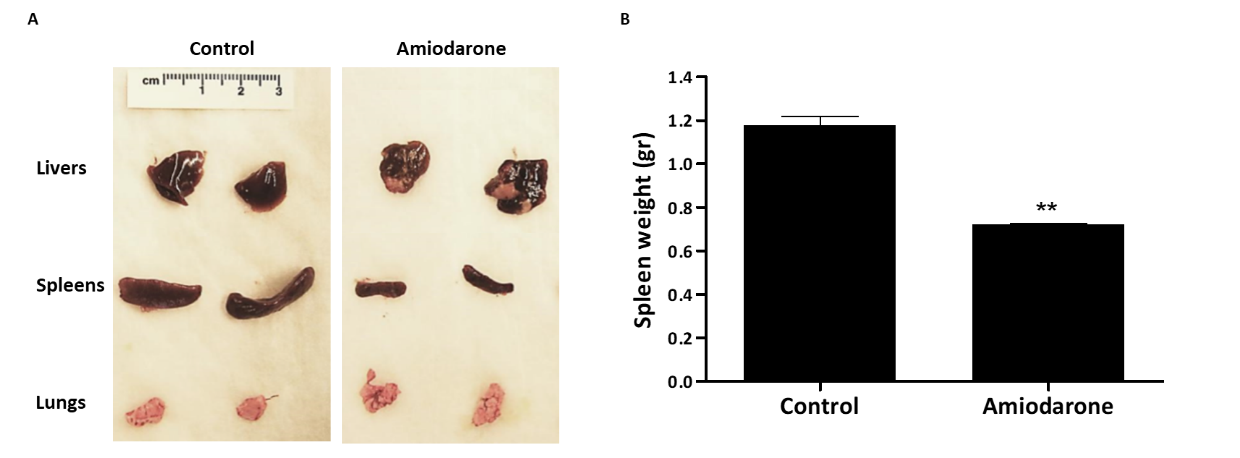


**Effect of Amiodarone in GBM tumor xenograft.**Foxn1 nu mice were injected S.C. with 5 x 10^6^ U-87 MG cells. When tumors reached an average volume of ~200 mm3, treatment with an I.P. injection of Amiodarone 0.1 mg/kg was initiated and administered daily. (A) Livers, spleens and lungs of control mice and Amiodarone-treated mice were harvested on day 14. (B) Measured weight of spleens from untreated mice compared with spleens from mice treated with Amiodarone. Objective lens 400x, *n*=3-5. ***p*<0.01, compared with non-treated control mice. Results are presented as mean ± SEM.


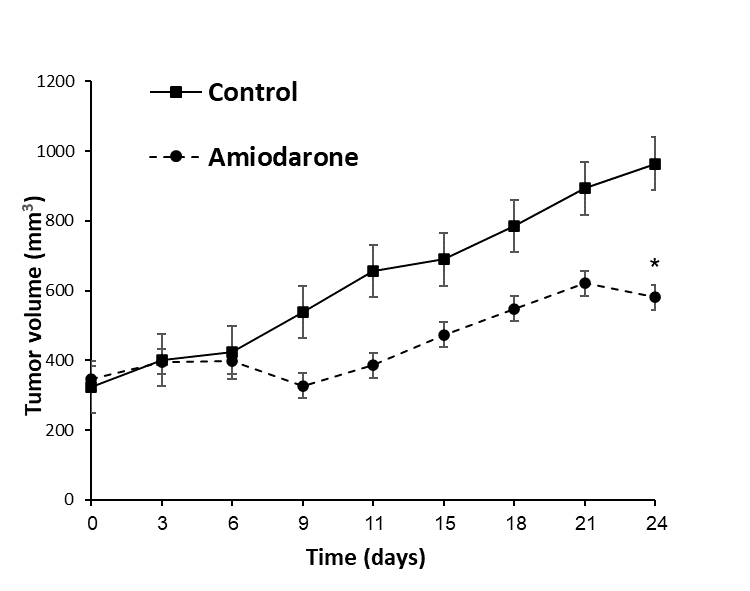
**Figure 7–figure supplement 2**

**Amiodarone reduces GBM tumor xenograft volume.**Foxn1 nu mice were injected S.C. with 5 x 10^6^ U-87 MG cells. When tumors reached an average volume of ~200 mm^3^, treatment with Amiodarone 0.05 mg/kg I.P. injection was initiated and administered daily. On day 24, tumor volumes of untreated mice compared with volumes of mice treated with Amiodarone were measured, *n*=5. **p*<0.05, compared with non-treated control mice. Results are presented as mean ± SEM.
